# 'ONE HEALTH' and parasitology

**DOI:** 10.1186/1756-3305-2-36

**Published:** 2009-08-12

**Authors:** Bruce Kaplan, Laura H Kahn, Thomas P Monath, Jack Woodall

**Affiliations:** 1 4748 Hamlets Grove Drive, Sarasota, Florida 34235, USA; 2 Program on Science and Global Security, Woodrow Wilson School of Public and International Affairs, Princeton University, 221 Nassau Street, 2nd floor, Princeton, New Jersey 08542, USA; 3 Kleiner Perkins Caufield & Byers, Pandemic & Biodefense Fund, 21 Finn Road, Harvard MA 01451, USA; 4 Nucleus for the Investigation of Emerging Infectious Diseases, Institute of Medical Biochemistry, Center for Health Sciences, Federal University, Rio de Janeiro, Brazil

## Editorial

Pertinent information about One Health may be accessed by visiting The One Health Initiative [1]. The website motto is "*One Health is the collaborative efforts of multiple disciplines working locally, nationally, and globally to attain optimal health for people, animals, plants and our environment*." The co-authors are a *pro bono *autonomous One Health team that has promoted the One Health concept on the website and elsewhere for several years.

One Health is a concept that proposes that a paradigm shift in approaching diseases of humans and animals is essential to meet the challenges of the 21^st ^century. Human and veterinary medicine, as well as all other scientific-health related disciplines, must begin forging co-equal, all-inclusive collaborations. Physicians, veterinarians, other health scientists, and their respective educational institutions, organizations and health agencies must work together. In the past, this approach has resulted in more rapid, efficacious, synergistic achievements in advancing health. Unfortunately, such an approach has been relatively rare during the 20^th ^century.

Since ancient times the concept that animal health and the environment influence human health has been around. One Health began in the late 19^th ^and 20^th ^centuries with physician leaders in medicine like Rudolf Virchow, known as the "Father of comparative medicine, cellular pathology, and veterinary pathology" and William Osler, called the "Father of Modern Medicine." They embraced the concept that human and animal health were inextricably linked. Virchow conducted experimental animal studies on *Trichinella spiralis *in porcine muscular tissue and cysticercosis and tuberculosis in cattle; he coined the term "zoonosis" and stated, "between animal and human medicine there are no dividing lines – nor should there be." Osler, who studied in Berlin with, and was influenced by Virchow, helped promote "One Health" as he taught veterinary pathology at Montreal Veterinary College, and established veterinary pathology as an academic discipline in North America.

The physician and veterinarian research team of Theobald Smith, and F. L. Kilborne, discovered the etiology of cattle fever, *Babesia bigemina*, and that it was transmitted by tick vectors, in 1893. Their important work helped set the stage for the discovery of the mosquito vector transmission of yellow fever by Walter Reed and colleagues.

In October 1976, the virus causing Ebola Hemorrhagic fever was identified and named by the U.S. Centers For Disease Control and Prevention (CDC) physician virologist, Karl Johnson, in collaboration with veterinarian pathologist-virologist, Fred Murphy. They collaborated on zoonotic viruses, their pathogenesis, epidemiology, and ecology for many years.

The late 20^th^, and particularly the early 21^st ^century, have been significantly subject to the risks from emerging deadly zoonotic diseases such as human immunodeficiency virus/acquired immune deficiency syndrome (AIDS), severe acute respiratory syndrome (SARS), West Nile virus and others. This phenomenon demands the urgent need for all human medical and veterinary medical scientific professionals to renew and increase collaborative efforts.

One Health has accelerated biomedical research discoveries and expanded scientific knowledge in clinical health care. Some clinical health examples are elaborated upon in the One Health Initiative website articles including cancer, orthopedic biomechanical prosthetics, diabetes, obesity, cardiovascular diseases, heart-valve advances, and vaccine development.

Many national and international organizations and governmental agencies have endorsed the One Health concept. Representatives from North America include the American Medical Association, American Veterinary Medical Association, American Society for Tropical Medicine and Hygiene, Association of American Medical Colleges, American Association of Veterinary Medical Colleges, the American Public Health Association, Association of Schools of Public Health, American Society of Microbiology, American College of Veterinary Microbiology, American Physiology Society, American Association of Veterinary Laboratory Diagnosticians, American Association of Wildlife Veterinarians, American Phytopathological Society and the Delta Society (re: human-animal bond phenomenon).

Globally, One Health supporters include the Immuno Valley Consortium in The Netherlands, Indian Veterinary Public Health Association, Croatian Society for Infectious Diseases, The Institute for Preventive Veterinary Medicine and Food Safety Lazio and Tuscany Regions Italy, Italian Society of Preventive Medicine, Corporation Red SPVet, Bogota, Colombia, Nigerian Veterinary Medical Association, Nigerian Biomedical and Life Sciences, The Global Alliance for Rabies Control, Society for Tropical Veterinary Medicine, The Institute of Tropical Medicine, Department of Animal Health, Antwerp, Belgium, and the World Association of Veterinary Laboratory Diagnosticians.

Significant One Health endorsement resolutions have been adopted by many of the above including the American Medical Association, American Veterinary Medical Association, American Society of Tropical Medicine and Hygiene, the Society for Tropical Veterinary Medicine and others.

The late renowned 20^th ^century veterinary epidemiologist, parasitologist, and global authority on zoonoses, Calvin W. Schwabe at the University of California coined the term "One Medicine" (now commonly referred to as "One Health") which was aimed at unifying human medical and veterinary medical disciplines against zoonotic diseases occurring in the public health arena. Two examples of recently emerging zoonotic disease epidemics include the avian influenza A H5N1 strain that primarily affects poultry (with some linked human occurrences) and the most recent human pandemic of H1N1 influenza that has spread across Asia, Africa, Europe and the United States.

Included among many current and previous online topics from *Parasites and Vectors *is the zoonotic protozoan parasite responsible for African Trypanosomiasis (sleeping sickness). An excellent history has been provided by Steverding [2]. An interesting common parasite of dogs, i.e. *Dirofilaria immitis *(heartworm) is also a rare zoonotic disease in humans. Aspects of this parasitic species and others are described by Otranto *et al. *[3].

Parasitologists, of all the health professional scientists, are generally most familiar with the long list of parasitic zoonoses that affect humans via animals as well as specific details pertaining to each. Hence, the critical need for One Health research collaborations and cooperation. Veterinary medical school students usually receive considerably more exposure to parasitology than do human medical school students. This is largely due to the much greater volume of endoparasite (and ectoparasite) infections and infection rates for animals vis-à-vis humans. Thus, epidemiologic prevention, diagnosis, control and treatment needs of animals exceed those for humans. Yet, there remain significant research requirements for understanding ideal management in human and animal species utilizing One Health principles.

## One Health in Action

The official report of the American Public Health Association's Control of Communicable Diseases Manual, 19^th ^Edition, edited by physician David L. Heymann, includes a staggering number of zoonotic parasites.

Heymann, a One Health supporter/advocate, is currently Chairman of the Health Protection Agency (HPA) with sites across the United Kingdom. He is also chairing an Australian scientific advisory committee for a One Health Symposium entitled, "The *1^st ^International One Health Congress: Human Health, Animal Health, the Environment and Global Survival*", tentatively proposed for 14–16 February 2011 in Melbourne. One Health supporter/advocate veterinarian, Martyn Jeggo Director, Australian Animal Health Laboratory (AAHL), chairs the organizing committee responsible for developing a framework for the symposium; AAHL will also be working in conjunction with a number of colleges.

In addition to African trypanosomiasis and dirofilariasis mentioned above, the global list of parasites of human concern includes amebiasis, angiostrongyliasis, anisakaiasis, ascariasis, babesiosis, balantidiasis, capillariasis, clonorchiasis, cryptosporidiosis, diphyllobothriasis, dracunculiasis, echinococcosis, giardiasis, hookworm disease, hymenolepiasis, leishmaniasis, malaria, shistosomiasis, strongyloidiasis, taeniasis, toxocariasis, toxoplasmosis, trichinellosis, and American trypanosomiasis (Chagas Disease).

In a press release of April 24, 2009, Sanaria Inc. announced that with support from the PATH Malaria Vaccine Initiative (MVI) it has initiated a Phase 1 clinical trial of its unique malaria vaccine candidate. Sanaria's approach "deploys a weakened form of the whole malaria parasite harvested from irradiated mosquitoes instead of small portions of the parasite." ... "While most malaria vaccines in clinical development consist of recombinant or genetically engineered proteins that represent small portions of the parasite, Sanaria's *Plasmodium falciparum *sporozoite vaccine candidate contains a weakened form of the entire malaria parasite." The Sanaria vaccine currently requires liquid nitrogen transport and storage, which poses challenges for widespread use in Africa.

An example of "One Health in Action" was a recent outreach to the veterinary medical community by Monath, one of the co-authors of this editorial in an effort to ascertain methods for vaccine storage and transport. The idea was to investigate and compare how veterinary vaccines were handled and if utilizing or incorporating some of these methods might be utilized and provide better transportation of the human malaria vaccines. A request for information on the distribution of veterinary vaccines using liquid nitrogen placed on the One Health Initiative website resulted in many potentially useful responses from industry and academia.

Medical professionals and health scientists must include parasitologists, microbiologists, physiologists, pathologist, physicians, osteopaths, veterinarians, dentists, nurses, biomedical engineers, physicists, biochemists, plant pathologists and others. Anyone capable of contributing should be considered important and co-equal without reservations.

One Health has indeed become the "Rosetta Stone" for a health enlightening paradigm shift revolution. It is the critical key that translates difficult problem solving into less difficult models. It presents a means for the health scientific communities to move towards a more panoramic view, a sustainable revolution (described as an environmental definition for our civilization to survive) and the pursuit of altruistic excellence, notwithstanding respectable status quo advancements of the past. One Health works!

## Competing interests

The authors declare that they have no competing interests.

## Authors' contributions

All authors contributed equally to this work
